# Hydrogen Sulfide: A Signal Molecule in Plant Cross-Adaptation

**DOI:** 10.3389/fpls.2016.01621

**Published:** 2016-10-26

**Authors:** Zhong-Guang Li, Xiong Min, Zhi-Hao Zhou

**Affiliations:** ^1^School of Life Sciences, Yunnan Normal UniversityKunming, China; ^2^Engineering Research Center of Sustainable Development and Utilization of Biomass Energy, Ministry of EducationKunming, China; ^3^Key Laboratory of Biomass Energy and Environmental Biotechnology, Yunnan Normal UniversityKunming, China

**Keywords:** cross-adaptation, hydrogen sulfide, signal crosstalk, stress tolerance

## Abstract

For a long time, hydrogen sulfide (H_2_S) has been considered as merely a toxic by product of cell metabolism, but nowadays is emerging as a novel gaseous signal molecule, which participates in seed germination, plant growth and development, as well as the acquisition of stress tolerance including cross-adaptation in plants. Cross-adaptation, widely existing in nature, is the phenomenon in which plants expose to a moderate stress can induce the resistance to other stresses. The mechanism of cross-adaptation is involved in a complex signal network consisting of many second messengers such as Ca^2+^, abscisic acid, hydrogen peroxide and nitric oxide, as well as their crosstalk. The cross-adaptation signaling is commonly triggered by moderate environmental stress or exogenous application of signal molecules or their donors, which in turn induces cross-adaptation by enhancing antioxidant system activity, accumulating osmolytes, synthesizing heat shock proteins, as well as maintaining ion and nutrient balance. In this review, based on the current knowledge on H_2_S and cross-adaptation in plant biology, H_2_S homeostasis in plant cells under normal growth conditions; H_2_S signaling triggered by abiotic stress; and H_2_S-induced cross-adaptation to heavy metal, salt, drought, cold, heat, and flooding stress were summarized, and concluded that H_2_S might be a candidate signal molecule in plant cross-adaptation. In addition, future research direction also has been proposed.

## Introduction

Cross-adaptation, widely existing in nature, is the phenomenon in which plants expose to a moderate stress can induce the resistance to other stresses (Li and Gong, 2011; Foyer et al., 2016; Hossain et al., 2016). For example, cold pretreatment can improve the heat tolerance of winter rye, salt shock can rapidly induce the cold tolerance in spinach and potato, ultraviolet radiation (UV-B) can enhance the heat tolerance in cucumber and the cold tolerance in *Rhododendron*, and mechanical stimulation can improve the heat tolerance and the chilling tolerance in tobacco cells (Knight, 2000; Li and Gong, 2011, 2013). Interestingly, Foyer et al. (2016) found that cross-adaptation also can be induced between abiotic and biotic stresses. Infection by mycorrhizal fungi can improve the resistance of tomato, sunflower, pea, and rice to drought, chilling, salinity, metal toxicity, and high temperature stress (Grover et al., 2011), while drought stress can reduce aphid fecundity in *Arabidopsis* (Pineda et al., 2016). Our previous work also showed that heat shock could improve the resistance of maize seedlings to heat, chilling, salt, and drought stress (Gong et al., 2001). Numerous studies found that the acquisition of stress tolerance including cross-adaptation was involved in a complex signal network consisting of many second messengers such as Ca^2+^, abscisic acid (ABA), hydrogen peroxide (H_2_O_2_) and nitric oxide (NO), as well as their crosstalk (Knight, 2000; Pandey, 2015; Li and Gu, 2016; Li and Jin, 2016; Niu and Liao, 2016; Wang et al., 2016). In tobacco, mechanical stimulation can successively trigger H_2_O_2_ and NO signaling (Li and Gong, 2011, 2013), heat shock can induce Ca^2+^ and ABA signaling one after the other (Gong et al., 1998a,b), which in turn induce cross-adaptation to heat and chilling stress, similar results were reported by Gong et al. (2001) in maize seedlings. These results indicate that the acquisition of cross-adaptation is involved in signal crosstalk among Ca^2+^, H_2_O_2_, NO, and ABA in plants. Recently, hydrogen sulfide (H_2_S) was also found to be a member of this signal network in plants (Calderwood and Kopriva, 2014; Hancock and Whiteman, 2014; Fotopoulos et al., 2015; Guo et al., 2016), indicating that H_2_S might be a signal molecule in plant cross-adaptation.

For a long time, H_2_S has been considered as merely a toxic intermediate of cell metabolism due to its strong affinity to Fe^2+^-containing proteins such as cytochrome oxidase, hemoglobin and myoglobin, which may have been primary cause of the mass extinction of species in the Permian (Li, 2013; Lisjak et al., 2013; Calderwood and Kopriva, 2014; Hancock and Whiteman, 2014; Fotopoulos et al., 2015; Guo et al., 2016; Yamasaki and Cohen, 2016). H_2_S can inhibit oxygen release from young seedlings of six rice cultivars (Bluebelle, Dawn, Norin 22, Saturn, Yubae, and Zenith) and nutrient uptake such as phosphorus (Li, 2013; Calderwood and Kopriva, 2014; Hancock and Whiteman, 2014). But nowadays, H_2_S is found to function as gaseous signal molecule at low concentration similar to carbon monoxide (CO) and NO in plants, and it has been shown that plants can actively synthesize endogenous H_2_S under normal, especially biotic or abiotic stress conditions (Li, 2013; Calderwood and Kopriva, 2014; Hancock and Whiteman, 2014; Yamasaki and Cohen, 2016). The accumulation of endogenous H_2_S has become a common response of plants to environmental stress, including salt, heavy metal (HM), drought, heat and cold stress, as well as pathogen infection, which may be closely associated with the acquisition of stress tolerance in plants (Li, 2013; Calderwood and Kopriva, 2014; Hancock and Whiteman, 2014). More interestingly, exogenously applied H_2_S, releasing from its donors such as NaHS and morpholin-4-ium 4-methoxyphenyl(morpholino) phosphinodithioate (GYY4137), shows significant positive effects on seed germination (Li et al., 2012a; Li and He, 2015; Wojtyla et al., 2016), organogenesis and growth (Lin et al., 2012; Fang T. et al., 2014), the regulation of senescence (Zhang et al., 2011), as well as the acquisition of stress tolerance such as salt (Christou et al., 2013), HM (Chen et al., 2013), drought (Christou et al., 2013), heat (Li et al., 2013a,b; Li, 2015c) and cold tolerance (Fu et al., 2013). These results indicate that H_2_S may be a candidate signal molecule in plant cross-adaptation. In addition, NaHS and GYY4137 are commonly used as H_2_S donors because they can release H_2_S when dissolved in water, but NaHS giving a relatively short burst of H_2_S, while GYY4137 giving a longer more prolonged exposure to H_2_S (Wang, 2012; Lisjak et al., 2013). However, whether H_2_S concentration in plant cells or tissues is consistent with that of NaHS and GYY4137 applied as well as actual H_2_S concentration triggering cross-adaptation need to be further investigated. In addition, H_2_S usually exist in the forms of H_2_S (approximately 20%) and HS^-^ (approximately 80%) in water solution, exact physiological concentration of H_2_S in plant cells or subcellular organelles is not clear.

Though, there are a lot of excellent reviews which expound potential physiological function of H_2_S in seed germination, plant growth and development, as well as the acquisition of stress tolerance (Li, 2013; Calderwood and Kopriva, 2014; Hancock and Whiteman, 2014, 2016; Jin and Pei, 2015; Guo et al., 2016; Scuffi et al., 2016; Yamasaki and Cohen, 2016), the role of H_2_S as a candidate signal molecule in plant cross-adaptation was not summarized in depth. Therefore, in this review, H_2_S homeostasis in plant cells under normal growth conditions, H_2_S signaling triggered by adverse environment and H_2_S-induced cross-adaptation to various abiotic stresses are summarized, which further uncovers that H_2_S may be a candidate signal molecule in plant cross-adaptation.

## H_2_S Homeostasis in Plant Cells

**FIGURE 1:**
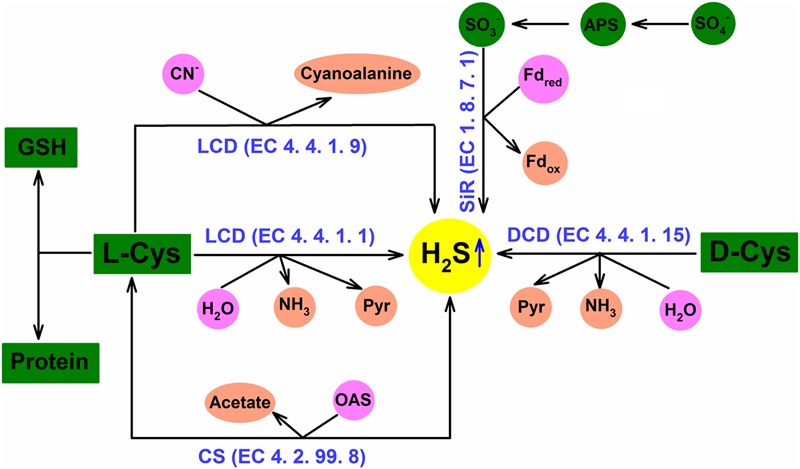
**Hydrogen sulfide (H_2_S) homeostasis in plant cells.** H_2_S homeostasis can be regulated by L-cysteine desulfhydrase (LCD), D-cysteine desulfhydrase (DCD), sulfite reductase (SiR), cyanoalanine synthase (CAS), and cysteine synthase (CS) pathways in plant cells (adapted from Li, 2015a).

As mentioned above, due to the dual role of H_2_S, that is, as cytotoxin at high concentration and as cell signal molecule at low concentration, H_2_S homeostasis in plant cells is very important to exert its physiological functions including cross-adaptation induction. In plant cells, there are many metabolic pathways to regulate H_2_S homeostasis, similar to other signal molecules like H_2_O_2_, NO. H_2_S homeostasis is closely regulated by L-cysteine desulfhydrase (LCD, EC 4.4.1.1), D-cysteine desulfhydrase (DCD, EC 4.4.1.15), sulfite reductase (SiR, EC 1.8.7.1), cyanoalanine synthase (CAS, EC 4.4.1.9), and cysteine synthase (CS, EC 4.2.99.8; Li, 2013, 2015a; **Figure 1**). LCD/DCD catalyzes the degradation of L-/D-cysteine to produce H_2_S, amine and pyruvate; SiR reduces sulfite to H_2_S using ferredoxin as electron donor; H_2_S can be released from cysteine in the present of hydrogen cyanide by CAS; CS, namely *O*-acetyl-(thiol)-serinelyase (OAS-TL), can incorporate H_2_S into *O*-acetyl-L-serine to form cysteine, and its reverse reaction can release H_2_S (Li, 2013, 2015a; **Figure 1**). Generally, plants synthesize H_2_S via LCD or DCD, which respond to environment stress and induce the acquisition of stress tolerance. In addition, excess H_2_S can be released to air (Li, 2013; Calderwood and Kopriva, 2014; Hancock and Whiteman, 2014).

## H_2_S Signaling Triggered By Abiotic Stress

**FIGURE 2:**
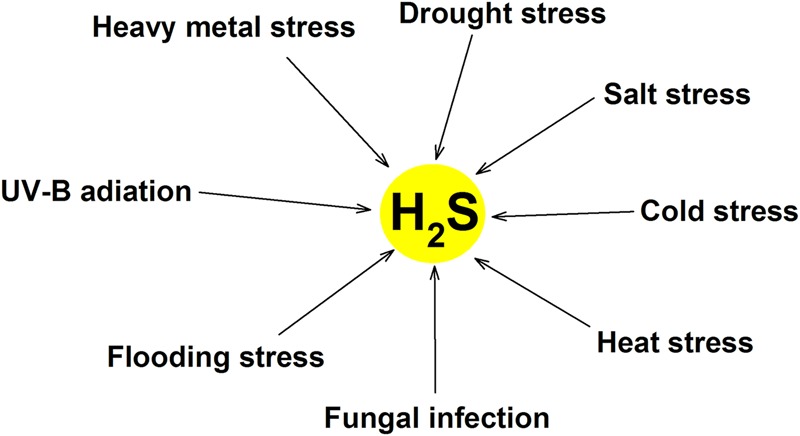
**Mutiple environmental stress can induce endogenous H_2_S production in plants.** Abiotic stress (heavy metal, drought, salt, cold, heat, flooding, and UV-B radiation) and biotic stress (fungal infection) induce the generation of endogenous H_2_S by mainly activating LCD.

Similar to other second messengers such as Ca^2+^, H_2_O_2_, ABA and NO, the rapid production of endogenous H_2_S in many species of plant can be triggered by numerous stresses (**Table 1**; **Figure 2**), this is a common response of plants to various abiotic stresses, which is closely associated with the acquisition of cross-adaptation in plants.

**Table 1:** Different abiotic stresses trigger endogenous H_2_S production in plants.

Species	Stress	H_2_S content		Reference
		Normal conditions	Stress conditions	
Rice	Cd	5 μmol g^-1^ FW	6 μmol g^-1^ FW	Mostofa et al., 2015
Chinese cabbage	Cd	0.38 nmol mg^-1^ Pr min^-1^	0.58 nmol mg^-1^ Pr min^-1^	Zhang et al., 2015
Foxtail millet	Cr^6+^	0.6 nmol mg^-1^ Pr min^-1^	1.6 nmol mg^-1^ Pr min^-1^	Fang H. et al., 2014
Alfalfa	NaCl	30 nmol g^-1^ FW	70 nmol g^-1^ FW	Lai et al., 2014
Strawberry	PEG-6000, NaCl	25 nmol g^-1^ FW	35 nmol g^-1^ FW	Christou et al., 2013
*Arabidopsis*	Drought	6 nmol mg^-1^ Pr min^-1^	14 nmol mg^-1^ Pr min^-1^	Jin et al., 2011
*Arabidopsis*	Cold	3 nmol g^-1^ FW	5 nmol g^-1^ FW	Shi et al., 2015
Grape	Cold	7 μmol g^-1^ FW	15 μmol g^-1^ FW	Fu et al., 2013
Bermudagrass	Cold	5 nmol g^-1^ FW	14 nmol g^-1^ FW	Shi et al., 2013
*Lamiophlomis rotata*	Cold	12 nmol g^-1^ FW	24 nmol g^-1^ FW	Ma et al., 2015
Tobacco	Heat	2 nmol g^-1^ FW	8 nmol g^-1^ FW	Chen et al., 2016
Barley	UV-B	125 nmol g^-1^ FW	230 nmol g^-1^ FW	Li et al., 2016
Pea	Hypoxia	0.8 μmol g^-1^ FW	1.5 μmol g^-1^ FW	Cheng et al., 2013

The FW and Pr in the table represent fresh weight and protein respectively.

### H_2_S Signaling Triggered by Heavy Metal Stress

The rapid production of H_2_S has become a common response of plants to various HM stress, among HMs, Cd is the most severe stress due to its toxicity and stability (Ahmad, 2016). In rice seedlings, 0.5 mM Cd stress resulted in an increment of H_2_S content from approximately 5 μmol g^-1^ fresh weight (FW) to approximately 6 μmol g^-1^ FW. The addition of 0.1 mM NaHS caused an even further increase in the level of H_2_S (approximately 8 μmol g^-1^ FW) as compared with Cd treatment alone. Exposure to 0.2 mM hypotaurine (HT, H_2_S scavenger) with NaHS decreased H_2_S level compared with NaHS alone, indicating that this elevated level of H_2_S is correlated with the enhanced Cd tolerance (Mostofa et al., 2015). Zhang et al. (2015) found that the endogenous H_2_S emission was stimulated by Cd stress in Chinese cabbage. The relative expression of DCD1 and DES1 (cysteine desulfhydrase, OAS-TL homogenous family) genes (responsible for H_2_S synthesis) was up-regulated after treatment with Cd with a range of concentrations (0, 5, 10, and 20 mM) for 24 h. Expression of DES1 at 5 mM Cd already showed a significant increase, and at 20 mM Cd was 4.7 times of the control. Following a similar pattern, the endogenous H_2_S concentrations also significantly rose from 0.38 to 0.58 nmol mg^-1^ protein min^-1^ at 20 mM. Chromium (Cr), existing in the form of Cr^3+^ and Cr^6+^, is regarded as the second most common HM, both forms have become major environmental pollution sources. In foxtail millet seedlings, Fang H. et al. (2014) also reported that the expressions of H_2_S-emission related genes LCD, DCD2, and DES markedly increased during the first 12 h of Cr^6+^ exposure following decline at 24 h, while the expression of DCD1 was consistently increased from 0 to 24 h under 10 mM Cr^6+^ stress. Additionally, the H_2_S production rate is induced by Cr^6+^ stress in dose- and time-dependent manner, and this induction was the most significant with 24 h of 10 mM Cr^6+^ treatment (from 0.6 to 1.6 nmol mg^-1^ protein min^-1^). These results imply that endogenous H_2_S synthesis was activated by Cr^6+^ stress by activating its emission system in foxtail millet. Inconceivably, in compared with to other plant species, the both species Chinese cabbage and foxtail millet show a remarkable tolerance to HM (Cd and Cr^6+^). At 20 mM Cd for Chinese cabbage and 10 mM Cr^6+^ for foxtail millet, these treatment concentrations are far beyond the physiological level (generally micromolar concentrations) for many plant species, the precise physiological, biochemical, and molecular mechanisms are waiting for being uncovered.

### H_2_S Signaling Triggered by Salt Stress

Salt stress commonly leads to an osmotic stress response, similar to drought stress, which triggers rapid generation of second messengers like H_2_S. In alfalfa seedlings, the increasing concentration of NaCl (from 50 to 300 mM) progressively caused the induction of total LCD activity and the increase of endogenous H_2_S production (from 30 to 70 nmol g^-1^ FW) (Lai et al., 2014). Exposure of strawberry seedlings to salinity (100 mM NaCl) and non-ionic osmotic stress (10% PEG-6000) greatly enhanced H_2_S concentration (48 and 50 nmol g^-1^ FW) in leaves, while 0.1 mM NaHS-pretreated plants subsequently exposed for 7 days to both stress factors were found to accumulate significantly higher amounts of H_2_S (55 nmol g^-1^ FW) in their leaves compared with NaCl-stressed plants (Christou et al., 2013).

### H_2_S Signaling Triggered by Drought Stress

One of the most severe abiotic stresses being experienced world-wide is drought. In *Arabidopsis* seedlings, the results of Shen et al. (2013) showed that treating wild type with polyethylene glycol (PEG) 8000, to simulate drought stress, caused an increase in production rate of endogenous H_2_S (0.8 nmol mg^-1^ protein min^-1^). At early stage of osmotic exposure (PEG 6000 for 2 days), the endogenous H_2_S in wheat seeds rapidly increased from 1.5 to 3.5 μmol g^-1^ dry weight (DW) (Zhang et al., 2010a).

### H_2_S Signaling Triggered by Low Temperature Stress

Low temperature is a major environmental stress factors that limit plant growth, development and distribution. In grape (*Vitis vinifera* L.) seedlings, chilling stress at 4°C induced the expression of L/DCD genes and increased the activities of L/DCD, which in turn enhanced endogenous H_2_S accumulation (from 7 to 15 μmol g^-1^ FW) (Fu et al., 2013). Similarly, Shi et al. (2013) also found that cold stress treatment at 4°C could induce the accumulation of endogenous H_2_S level (14 nmol g^-1^ FW) in bermudagrass [*Cynodon dactylon* (L). Pers.] seedlings. To uncover the adaptive strategies of alpine plants to the extremely cold conditions prevailing at high altitudes, Ma et al. (2015), using a comparative proteomics, investigated the dynamic patterns of protein expression in *Lamiophlomis rotata* plants grown at three different altitudes (4350, 4,800, and 5,200 m), and the results showed that the levels and enzyme activities of proteins (OAS-TL, CAS, L/DCD) involved in H_2_S biosynthesis markedly increased at higher altitudes (4,800 and 5,200 m), and that H_2_S accumulation increased to 12, 22, and 24 nmol g^-1^ FW, respectively, demonstrating that H_2_S plays a central role during the adaptation of *L. rotata* to environmental stress at higher altitudes.

### H_2_S Signaling Triggered by High Temperature Stress

Similar to other stresses, high temperature also can induce endogenous H_2_S generation in many species of plant. In 3-week-old seedlings of tobacco, Chen et al. (2016) found that treatment with high temperature at 35°C increased the activity of LCD, which in turn induced the production of endogenous H_2_S (8 nmol g^-1^ FW) in tobacco seedlings, and that H_2_S production remained elevated level after 3 days of high temperature exposure. More interestingly, H_2_S production by high temperature can induce the accumulation of jasmonic acid, followed by promoting nicotine synthesis. These data suggest that H_2_S and nicotine biosynthesis is linked in tobacco plants subjected to high temperature stress. Additionally, heat stress caused a marked modulation in H_2_S content in strawberry seedlings, as indicated in a significant increase after 1, 4, and 8 h of exposure to 42°C compared with control plants. A significant increase in H_2_S content was also observed in 0.1 mM NaHS-pretreated plants after 1 h exposure to heat stress, gradually lowering to control levels thereafter (Christou et al., 2014).

### H_2_S Signaling Triggered by UV-B Radiation

Recently, Li et al. (2016) found that UV-B radiation could induce H_2_S production in leaves of barley seedlings, reaching a peak of approximately 230 nmol g*^-^*^1^ FW after 12 h of exposure, which in turn promoted the accumulation of UV-absorbing compounds flavonoids and anthocyanins. H_2_S began to decline with time, but it is overall significantly higher than that of the control (approximately 125 nmol g^-1^ FW) at 48 h of exposure. A similar trend was observed for LCD activity, which was corroborated by the application of DL-propargylglycine (PAG, an inhibitor of LCD) that resulted in complete inhibition of the H_2_S production and the accumulation of UV-absorbing compounds induced by UV-B radiation (Li et al., 2016).

### H_2_S Signaling Triggered by Hypoxia and Fungal Infection

Flooding often leads to hypoxia in plant roots, which significantly limits agriculture production. In pea (*Pisum sativum* L.) seedlings, Cheng et al. (2013) found that hypoxia could activate H_2_S biosynthesis system (LCD, DCD, OAS-TL, and CS), which in turn induced the accumulation of endogenous H_2_S from approximately 0.9 (control) to 5.1 μmol g^-1^ FW (hypoxia for 24 h), indicating that H_2_S might be a hypoxia signaling that triggers the tolerance of the pea seedlings to hypoxic stress, this hypothesis was further supported by exogenously applied NaHS.

Pathogen infection is a common biotic stress in plants. In oilseed rape (*Brassica napus* L.) seedlings, fungal infection with *Sclerotinia sclerotiorum* led to an even stronger increase in H_2_S, reaching a maximum of 3.25 nmol g^-1^ DW min^-1^ 2 days after infection, suggesting that the release of H_2_S seems to be part of the response to fungal infection (Bloem et al., 2012).

### H_2_S Signaling Triggered by Exogenously Applied NaHS or Up-regulating the Expression of L/DCD

In addition to above-described abiotic and biotic stressors, H_2_S signaling in plant cells also can be triggered by exogenously applying NaHS (H_2_S donor) or up-regulating the expression of genes involved in H_2_S biosynthesis like L/DCD under normal growth conditions. In strawberry seedlings, treatment of root with 0.1 mM NaHS resulted in significantly elevated H_2_S concentration (35 nmol g^-1^ FW) in leaves compared with control plants (25 nmol g^-1^ FW) (Christou et al., 2013). In wheat seeds, the endogenous H_2_S level [4.5 μmol g^-1^ dry weight (DW)] in NaHS-treated seed was slightly higher than that of control (1.7 mol g^-1^ DW) (Zhang et al., 2010a). These results indicated that H_2_S is easy to enter into plant cells and follow on being transported to other tissues or organs due to its highly lipophilic property, which in turn exert its physiological role in plants.

Additionally, Jin et al. (2011) found that the *Arabidopsis* seedlings expressing L/DCD showed higher endogenous H_2_S content under both normal (6 nmol mg^-1^ protein min^-1^) and drought stress conditions (14 nmol mg^-1^ protein min^-1^) compared with the control (3 nmol mg^-1^ protein min^-1^), and the expression pattern of L/DCD was similar to the drought associated genes dehydration-responsive element-binding proteins (*DREB2A, DREB2B, CBF4, and RD29A*) induced by dehydration, while exogenous application of H_2_S (80 μM) was also found to stimulate further the expression of drought associated genes. In addition, drought stress significantly induced endogenous H_2_S production in both transgenetic plant and wild type, a process that was reversed by re-watering (Jin et al., 2011). Interestingly, *Arabidopsis* seedlings overexpressing LCD or pre-treated with NaHS exhibited higher endogenous H_2_S level (from 2 to 10 nmol g^-1^ FW), followed by improving abiotic stress (drought, salt, and chilling) tolerance and biotic stress (bacteria) resistance, while LCD knockdown plants or HT (H_2_S scavenger) pre-treated plants displayed lower endogenous H_2_S level and decreased stress resistance (Shi et al., 2015).

In conclusion, above-mentioned researches in this section display that: (1) under normal growth conditions, the content of endogenous H_2_S or production rate in various plant species are different, ranging from 2 nmol g^-1^ FW to 7 μmol g^-1^ FW or 0.38 to 6 nmol mg^-1^ protein min^-1^. These differences may be relative to measurement methods, plant species and development stage, and experiment system. (2) Under abiotic stress conditions, the level of endogenous H_2_S in various plant species is averagely increased by 2∼2.5-fold, indicating that different environment stresses can trigger the H_2_S signaling, which may be a trigger that induces the acquisition of cross-adaptation in plants.

## H_2_S-Induced Cross-Adaptation

**FIGURE 3:**
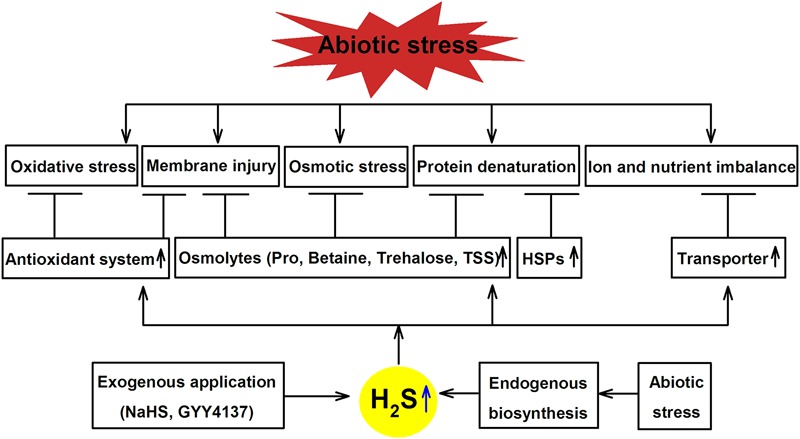
**Mechanisms underlining H_2_S-induced abiotic tolerance in plants.** Abiotic stress causes oxidative stress, membrane injury, osmotic stress, protein denaturation, as well as ion and nutrient imbalance, while exogenously applied or endogenously synthesized H_2_S can alleviate these damages by enhancing the activity of antioxidant system, synthesizing osmolytes and heat shock proteins (HSPs) and regulating ion and nutrient balance (adapted from Min et al., 2016).

As described above, not only there are a broad range of environmental stressors can trigger H_2_S signaling in plants, but pretreating plants with exogenously applied H_2_S can provide additional resistance to subsequent stress exposure. The next section explores the role of H_2_S as an important signaling molecule for cross-adaptation to HM, salt, drought, cold, heat and flooding stress by enhancing antioxidant system activity, accumulating osmolyte, synthesizing heat shock proteins (HSPs), as well as maintaining ion and nutrient balance (**Table 2**; **Figure 3**), which may be common mechanism of cross-adaptation induced by H_2_S.

**Table 2:** NaHS (H_2_S donor)-induced cross-adaptation in plants.

Species	Tolerance	NaHS (mM)	Responsible factors	Reference
Pea	As	0.1	AsA–GSH cycle, reducing As accumulation	Singh et al., 2015
Wheat	Cr	1.2	Activating antioxidant enzymes	Zhang et al., 2010b
Wheat	Cu	1.4	Promoting amylase and esterase activities, maintain plasma membrane integrity	Zhang et al., 2008
Wheat	Al	0.6	Decreasing Al accumulation, alleviating citrate secretion, and oxidative stress	Zhang et al., 2010c
Barley	Al	0.2	Decreasing Al accumulation, alleviating citrate secretion, and oxidative stress	Chen et al., 2013
*Solanum nigrum*	Zn	0.2	Enhancing the metallothioneins, alleviating oxidative stress, reducing Zn uptake	Liu et al., 2016
Wheat	Salt	0.05	Promoting amylase and esterase activities	Bao et al., 2011
Alfalfa	Salt	0.1	Activating antioxidant enzyme	Wang et al., 2012
*Arabidopsis*	Salt	0.2	Maintaining a lower Na^+^/K^+^ ratio, promoting the genes expression and the phosphorylation of H^+^-ATPase and Na^+^/H^+^ antiporter	Li J. et al., 2014
Wheat	PEG-6000	0.6	Increasing CAT and APX activities, reducing lipoxygenase activity	Zhang et al., 2010d
Wheat	PEG-6000	1.0	Increased antioxidant enzymes activities and gamma-glutamylcysteine synthetase	Shan et al., 2011
*Arabidopsis*	Drought	0.08	Stimulating the expression of drought associated genes	Jin et al., 2011
*Vicia faba*	Drought	0.1	Increasing relative water content	García-Mata and Lamattina, 2010
Bermudagrass	Cold	0.5	Modulating antioxidant enzymes and non-enzymatic antioxidant	Shi et al., 2013
Grape	Cold	0.1	Enhancing SOD activity and the expression of VvICE1 and VvCBF3 genes	Fu et al., 2013
*Arabidopsis*	Cold	0.1	Up-regulating the transcripts of multiple abiotic and biotic stress-related genes	Shi et al., 2015
*Lamiophlomis rotata*	Cold	0.05	Increasing antioxidant enzyme activity, proline and sugar accumulation	Ma et al., 2015
Banana	Cold	0.5	Increasing the phenylalanine ammonia lyase activity, total phenolics content and antioxidant capacity	Luo et al., 2015
Strawberry	Heat	0.1	Maintaining ascorbate/glutathione homeostasis, inducting gene expression of enzymatic antioxidants, HSPs and aquaporins	Christou et al., 2014
Maize	Heat	0.7	Increasing antioxidant activity	Li Z.G. et al., 2014
Maize	Heat	0.5	Inducing proline accumulation	Li and Gong, 2013; Li et al., 2013a
Tobacco	Heat	0.05	Increasing antioxidant activity	Li et al., 2012b, 2015
Pea	Hypoxia	0.1	Protecting ROS damage, inhibiting ethylene production	Cheng et al., 2013

### H_2_S-Induced Metal and Metalloid Tolerance

Heavy metals refer to a group of metal elements with a density greater than 6 g/cm^3^, including Cr, Cu, Zn, and so forth (Gupta et al., 2013; Ahmad, 2016). Due to their toxicity and stablility, HM has become the major abiotic stress in plants, and even threatens human health by way of the food chain. HM stress commonly results in oxidative stress, that is, the excessive accumulation of ROS, which leads to lipid peroxidation, protein oxidation, enzyme inactivation, and DNA damage (Yadav, 2010; Gupta et al., 2013; Ahmad, 2016). However, higher plants have evolved a sophisticated antioxidant defense system to scavenge excessive ROS and maintain its homeostasis in plants (Foyer and Noctor, 2009, 2011).

Arsenic (As) is a highly toxic metalloid, it is major pollutant in the soil. In pea seedlings, As treatment increased the accumulation of ROS, which in turn damage to lipids, proteins and biomembranes. Meanwhile, higher cysteine level was observed in As-stress seedlings in comparison to all other treatments (As-free; NaHS; As + NaHS), while these effects were alleviated by the addition of NaHS (Singh et al., 2015). Further experiments showed that As treatment inhibited the activity of the enzymes involved in the ascorbic acid (AsA)–glutathione (GSH) cycle, whereas their activities were enhanced by application of NaHS (Singh et al., 2015). In addition, the redox status of AsA and GSH was disturbed, as indicated by a steep decline in their reduced/oxidized ratios. However, exogenously applied NaHS restored the redox status of the AsA and GSH pools under As stress (Singh et al., 2015). Furthermore, NaHS treatment ameliorated As toxicity, which was coincided with the increased accumulation of H_2_S. The results demonstrated that H_2_S might counterbalance ROS-mediated damage to macromolecules by reducing the accumulation of As and triggering up-regulation of the AsA–GSH cycle, further suggesting that H_2_S plays a crucial role in plant priming, and in particular for pea seedlings in mitigating As stress.

Under Cr stress, exogenous application of NaHS could improve the germination rate of wheat seeds in a dose-dependent manner and the activities of amylase, esterase as well as antioxidant enzymes superoxide dismutase (SOD), catalase (CAT), ascorbate peroxidase (APX) and glutathione peroxidase (GPX), whereas reduced the activity of lipoxygenase and over-production of malondialdehyde (MDA) as well as H_2_O_2_ induced by Cr, and sustained higher endogenous H_2_S level (Zhang et al., 2010b). Additionally, NaHS pretreatment increased the activities of SOD and CAT, but decreased that of lipoxygenase in wheat under Cu stress (Zhang et al., 2008), these results were consisted with the response of wheat to Cr stress (Zhang et al., 2010b).

Also, NaHS could alleviate the inhibitory effect of Cu stress in wheat in a dose-dependent manner, and H_2_S or HS^-^ derived from NaHS rather than other sulfur-containing components (S^2-^, SO_4_^2-^, SO3^2-^, HSO_4_^-^, and HSO_3_^-^) attribute to the potential role in promoting seed germination under Cu stress (Zhang et al., 2008). Further experiments showed that NaHS could increase amylase and esterase activities, reduced the disturbance of plasma membrane integrity induced by Cu in the radicle tips, and sustain lower MDA and H_2_O_2_ levels in germinating seeds (Zhang et al., 2008), similar to the reports by (Zhang et al., 2010b).

Aluminium (Al), a non-essential element for plants, adversely affects plant growth, development and survival, especially in acid soil. In barley (*Hordeum vulgare* L.) seedlings, Al stress inhibited the elongation of roots, while pretreatment with NaHS partially rescued the inhibition of root elongation induced by Al, and this rescue was closely correlated with the decrease of Al accumulation in seedlings (Chen et al., 2013). Additionally, application of NaHS significantly alleviated citrate secretion and oxidative stress (as indicated in lipid peroxidation as well as ROS burst) induced by Al by activating the antioxidant system (Chen et al., 2013). Similar results were reported by Zhang et al. (2010c) in wheat (*Triticum aestivum* L.).

Though zinc (Zn) is an essential element for plants, its toxic effects can be observed when being excessive accumulation in plants. In *Solanum nigrum* L. seedlings, H_2_S ameliorated the inhibition of growth by excess Zn, especially in roots, and an increase in free cytosolic Zn^2+^ content in roots, which was correlated well with the down-regulation of Zn uptake and homeostasis related genes expression like zinc-regulated transporter (ZRT), iron-regulated transporter (IRT)-like protein (ZIP) and natural resistance associated macrophage protein (NRAMP) (Liu et al., 2016). In addition, H_2_S further enhanced the expression of the metallothioneins to chelate excessive Zn and alleviated Zn-oxidative stress by regulating the genes expression of antioxidant enzymes (Liu et al., 2016).

### H_2_S-Induced Salt Tolerance

Salts stress is negative effects of excessive salt on seed germination, plant growth and development, and even survival, which is a major abiotic stress in agriculture production world-wide. Salt stress commonly leads to direct and indirect injury, namely ion toxicity, osmotic stress, nutrient imbalance, and oxidative stress (Ahmad et al., 2013a,b). To combat with salt injury, plants have evolved many protective strategies, including osmotic adjustment by synthesizing osmolytes such as proline (Pro), glycine betaine (GB), trehalose (Tre), and total soluble sugar (TSS); ion and nutrient balance by regulating transporter; and enhancement of antioxidant capacity by activating the activity of antioxidant enzymes SOD, CAT, APX, GPX and glutathione reductase (GR), as well as by synthesizing antioxidants like AsA and GSH (Ahmad et al., 2013a,b). In salt-sensitive wheat cultivar LM15, the results of Bao et al. (2011) showed that wheat seed priming with different concentrations of NaHS (0.01, 0.05, 0.09, 0.13 mM) for 12 h could significantly alleviate the inhibition of seed germination and seedling growth induced by 100 mM NaCl in a concentration-dependent manner, as indicated in germination rate, germination index, vigor index and growth of seedlings of wheat. In alfalfa (*Medicago sativa*), NaHS pretreatment differentially activate total and isoenzymatic activities as well as corresponding transcripts of antioxidant enzymes (SOD, CAT, POD, and APX) under 100 mM NaCl stress, thus resulting in the alleviation of oxidative damage induced by NaCl (Wang et al., 2012). In addition, NaCl stress inhibited seed germination and seedling growth, but pretreatment with NaHS could significantly attenuate this inhibitive effect and increase the ratio of potassium (K) to sodium (Na) in the root parts (Wang et al., 2012). Also, under 100 mM NaCl stress, *Arabidopsis* roots displayed a great increase in electrolyte leakage and Na^+^/K^+^ ratio, indicating that *Arabidopsis* was sensitive to salt stress, while treatment with NaHS enhanced the salt tolerance by maintaining a higher K^+^/Na^+^ ratio (Li J. et al., 2014). In addition, the level of gene expression and the phosphorylation of plasma membrane H^+^-ATPase and Na^+^/H^+^ antiporter protein was promoted by H_2_S, while the effect of H_2_S on the plasma membrane Na^+^/H^+^ antiporter system was removed by diphenylene iodonium (DPI, a PM NADPH oxidase inhibitor) or dimethylthiourea (DMTU, an ROS scavenger) (Li J. et al., 2014), suggesting that H_2_S can maintain ion homeostasis in salt-stress *Arabidopsis* root in the H_2_O_2_-dependent manner.

### H_2_S-Induced Drought Tolerance

Similar to other stressors, drought stress, namely water deficiency, usually leads to osmotic stress and oxidative stress, which adversely affects plant growth, development and production (Iqbal et al., 2016). Plants can maintain water balance and ROS homeostasis by osmotic adjustment and antioxidant system (Foyer and Noctor, 2009, 2011; Iqbal et al., 2016). Zhang et al. (2010d) found that the germination rate reduced gradually with the increasing concentrations of PEG-6000, which mimicked osmotic stress, while NaHS treatment could promote wheat seed germination under osmotic stress in a dose-dependent manner, Na^+^ and other sulfur-containing components (S^2-^, SO_4_^2-^, SO3^2-^, HSO_4_^-^, and HSO_3_^-^) were not able to replace NaHS, confirming H_2_S or HS^-^ derived from NaHS contribute to the protective roles (Zhang et al., 2010d). Further experiments showed that NaHS treatment significantly increased CAT and APX activities, reduced that of lipoxygenase as well as the accumulation of MDA and H_2_O_2_ in seeds (Zhang et al., 2010d). Additionally, exogenously applied NaHS increased the activities of APX, GR, dehydroascorbate reductase (DHAR) and gamma-glutamylcysteine synthetase in wheat seedlings, as well as the contents of AsA, GSH, total ascorbate and total glutathione under water stress compared to the control without NaHS treatment, which in turn decreased the MDA content and electrolyte leakage induced by water deficiency in wheat seedlings (Shan et al., 2011). In *Arabidopsis* seedlings, under drought stress, the expression pattern of L/DCD was similar to the drought associated genes, whose express was stimulated further by H_2_S (Jin et al., 2011). Also, seedlings treated with NaHS exhibited a higher survival rate and a significant reduction in the size of the stomatal aperture compared to the control (Jin et al., 2011). In addition to these, García-Mata and Lamattina (2010) also found that, in *Vicia faba* (L.) var. major and *Impatiens walleriana* Hook. f., H_2_S treatment could increase relative water content (RWC) and protect plants against drought stress.

### H_2_S-Induced Cold Tolerance

Low temperature stress includes chilling stress (>0°C) and freezing stress (<0°C). Low temperature usually leads to osmotic stress and oxidative stress, plants can reduce the low temperature injury by osmotic adjustment and activating antioxidant system (Foyer and Noctor, 2009, 2011; Iqbal et al., 2016). Shi et al. (2013) found that exogenous application of NaHS conferred multiple stress tolerance including freezing tolerances in bermudagrass, in reflected in decreased electrolyte leakage and increased survival rate under freezing conditions. Additionally, NaHS treatment mitigated the ROS burst and cell damage induced by freezing stress via modulating the activities of antioxidant enzymes CAT, GPX and GR, as well as non-enzymatic GSH pool and redox state (Shi et al., 2013). In grape (*Vitis vinifera* L) seedlings, Fu et al. (2013) reported that treatment with NaHS showed the high activity of SOD and gene expression of VvICE1 and VvCBF3, lowed superoxide radical and MDA levels as well as cell membrane permeability under chilling stress at 4°C, while HT treatment displayed contrary effect under the chilling stress. Also, *Arabidopsis* seedlings overexpressing LCD or pretreating with NaHS exhibited higher endogenous H_2_S level and stronger chilling stress tolerance, while LCD knockdown or HT pre-treated plants displayed lower endogenous H_2_S level and weaker stress resistance. Moreover, H_2_S could up-regulate the expression of genes involved in multiple abiotic and biotic stress and inhibited ROS accumulation (Shi et al., 2015). Ma et al. (2015) found that the levels and enzyme activities of proteins involved in H_2_S biosynthesis (L/DCD, CAS, OAS-TL) markedly increased at higher altitudes at 4800 and 5200 m, which in turn maintained higher H_2_S level. Exogenous H_2_S application reduced ROS and RNS (reactive nitrogen species) damage by increasing antioxidant enzyme and GSNOR (*S*-nitrosoglutathione reductase) activities, activated the downstream defense response, resulting in protein degradation as well as Pro and SS accumulation. However, such defense responses could be reversed by HT and PAG, respectively. These results illustrated that H_2_S plays a central role in *L. rotata* uses multiple strategies to adapt to the alpine stress environment. Also, H_2_S fumigation maintained higher values of lightness and peel firmness of banana fruit and reduced the accumulation of MDA under chilling stress (Luo et al., 2015). In addition, H_2_S could increase the activities of GPX, SOD, CAT, APX, GR and the phenylalanine ammonia lyase and total phenolics content, which in turn improved antioxidant capacity of banana fruits, reducing H_2_O_2_ and superoxide anion accumulation (Luo et al., 2015). Further experiments also found that H_2_S fumigation elevated Pro content by activating P5CS activity and decreasing that of ProDH, which might be related to chilling injury tolerance improvement (Luo et al., 2015), similar to the report by Li and Gong (2013). These data indicate that H_2_S alleviated the chilling injury may be achieved through the enhancement of antioxidant system and Pro accumulation in banana fruit.

### H_2_S-Induced Heat Tolerance

Along with global warming, high temperature has already become a noticeable abiotic stress worldwide, and the mechanisms of high temperature injury and heat tolerance have attracted much attention (Wahid et al., 2007; Asthir, 2015; Hemmati et al., 2015). Christou et al. (2011) found that pre-treatment of roots with NaHS effectively alleviated the decrease in leaf chlorophyll fluorescence, stomatal conductance and relative leaf water content in strawberry (*Fragaria x ananassa* cv. Camarosa) under heat stress at 42°C, as well as an increase in ion leakage and MDA accumulation in comparison with plants directly subjected to heat stress. In addition, NaHS pretreatment preserved AsA/GSH homeostasis, as evidenced by lower AsA and GSH pool redox disturbances and enhanced transcription of AsA and GSH biosynthetic enzymes, 8 h after heat stress exposure. Furthermore, NaHS root pretreatment increased the gene expression of antioxidant enzymes (cAPX, CAT, MnSOD, GR), heat shock proteins (HSP70, HSP80, HSP90), and aquaporins (PIP) (Christou et al., 2014). These results suggest that H_2_S root pretreatment activates a coordinated network of heat shock defense-related pathways at a transcriptional level and systemically protects strawberry plants from heat stress-induced damage. Our previous study also showed that 0.7 mM NaHS treatment increased the activities of CAT, GPX, SOD and GR, and the contents of GSH and AsA, as well as the ratio of reduced antioxidants to total antioxidants [AsA/(AsA+DHA) and GSH/(GSH +GSSG)] in maize seedlings under normal culture conditions compared with the control (Li Z.G. et al., 2014). Under heat stress, antioxidant enzymes activities, antioxidants contents and the ratio of the reduced antioxidants to total antioxidants in control and treated seedlings all decreased, but NaHS-treated seedlings maintained higher antioxidant enzymes activities and antioxidants levels as well as reduced antioxidants/total antioxidants ratio (Li Z.G. et al., 2014), similar results also were found in tobacco cells (Li et al., 2015). In addition, NaHS pretreatment significantly increased the survival percentage of tobacco cells under heat stress and regrowth ability after heat stress, alleviated a decrease in vitality of cells and an increase in electrolyte leakage and MDA accumulation (Li et al., 2012b). Meanwhile, the heat tolerance induced by NaHS was markedly enhanced by exogenous application of Ca^2+^ and its ionophore A23187, respectively, while was weakened by addition of Ca^2+^ chelator ethylene glycol-bis(*b*-aminoethylether)- *N,N,N′,N*′-tetraacetic acid, plasma membrane channel blocker La^3+^, as well as calmodulin antagonists chlorpromazine and trifluoperazine, respectively (Li et al., 2012b). Similarly, in maize, pretreatment with NaHS markedly improved the germination percentage of seeds and the survival percentage of seedlings under heat stress, alleviated an increase in electrolyte leakage of roots and a decrease in tissue vitality and accumulation of MDA in coleoptiles of maize seedlings (Li et al., 2013a). Furthermore, NaHS pretreatment could improve the activity of Δ^1^-pyrroline-5-carboxylate synthetase (P5CS) and lowered that of Pro dehydrogenase (ProDH), which in turn induced the accumulation of endogenous Pro in maize seedlings (Li et al., 2013a). Also, exogenously applied Pro could increase endogenous Pro content, followed by increase in the survival percentage of maize seedlings under heat stress (Li et al., 2013a). These results suggest that NaHS pretreatment can improve the heat tolerance in plants and the acquisition of heat tolerance induced by NaHS may require the synergistic effect of antioxidant system, calcium messenger system, HSPs and Pro.

### H_2_S-Induced Flooding Tolerance and Pathogen Resistance

Flooding stress usually causes hypoxia, and even anoxia in plant roots, plants can improve hypoxia tolerance by reducing oxidative damage (van Dongen and Licausi, 2014). Cheng et al. (2013) found that hypoxia could induce root tip death of pea seedlings, while pretreatment with exogenous H_2_S dramatically alleviated cell death by protecting root tip cell membranes from ROS damage induced by hypoxia and by inhibiting ethylene production. Conversely, root tip death induced by hypoxia was strongly enhanced by inhibiting the key enzymes responsible for endogenous H_2_S biosynthesis (adding hydroxylamine to inhibit LCD activity). These results demonstrated that H_2_S can enhance the tolerance of the plant to hypoxic stress by alleviating hypoxia-induced root tip death in pea seedlings.

More interestingly, H_2_S also could transcriptionally regulate MIR393-mediate auxin signaling, including MIR393a/b and their target genes (TIR1, AFB1, AFB2, and AFB3), and this regulation was related with H_2_S-induced antibacterial resistance (Shi et al., 2015).

All of the above studies in this section show exogenous application of NaHS (a H_2_S donor) can induce cross-adaptation to HM, salt, osmosis, drought, cold, heat and hypoxia stresses in different plant species, and the optimal NaHS concentration range from 0.05 to 1.5 mM (**Table 2**), while higher NaHS concentration (>1.5 mM) exhibits negative effect on plant growth, development, survival, and even the acquisition of stress tolerance. Therefore, the optimal concentration of NaHS should be carefully selected according to plant species and experimental system.

## Conclusion and Future Prospective

In general, after undergoing a moderate stress, plants not only can improve the resistance to this stress, but also can increase the tolerance to subsequent other stresses, which known as cross-adaptation. Many studies found that signaling triggered by a moderate stress, such as Ca^2+^, ABA, H_2_O_2_, and NO signaling, is a common response of plants to abiotic and biotic stress, which in turn induces the acquisition of cross-adaptation. In addition, exogenously applied these signal molecules also can trigger corresponding signaling, followed by improving stress tolerance of plants, thus Ca^2+^, ABA, H_2_O_2_, and NO are considered to be candidate signal molecules in cross-adaptation in plants (Knight, 2000; Gong et al., 2001; Li and Gong, 2011; Li et al., 2012b; Fang H. et al., 2014; Qiao et al., 2015; Chen et al., 2016). More recently, many research groups found that a number of abiotic stresses also can trigger H_2_S signaling, while exogenously applied H_2_S can induce cross-adaptation to multiple stresses, indicating that H_2_S represents a potential candidate signal molecule in cross-adaptation in plants (Li, 2013; Lisjak et al., 2013; Calderwood and Kopriva, 2014; Hancock and Whiteman, 2014; Fotopoulos et al., 2015; Guo et al., 2016). However, H_2_S acts as a signal molecule in plants cross-adaptation, the following questions need to be further answered: (1) Receptor or target of H_2_S. Due to H_2_S is easy to penetrate the cell membrane, maybe there is no H_2_S receptor in plant cells, but Li et al. (2011) and Aroca et al. (2015) found that H_2_S could modify the activity of some proteins with sulfhydryl (-SH) by sulfhydrylation (-SSH), whether these proteins are the receptors of H_2_S needs to be further research. (2) Physiological concentration of H_2_S. Many assay methods for H_2_S including colorimetric, fluorescence-based, gas chromatographic and electrochemical methods give highly contrasting results (**Table 2**; Peng et al., 2012; Li, 2015b), so accurate physiological concentration of H_2_S in plant cells or organelles is waiting for uncovering. It will be important to design the stress treatments closer to physiologically relevant stress intensities, thus low micromolar rather than millimolar HM concentration should be investigated in order to strengthen the conclusions. (3) Crosstalk between H_2_S and other signal molecules in cross-adaptation. The acquisition of abiotic tolerance is involved in a signal network consisting of many signal molecules including H_2_S, interaction among signal molecules needs to be updated and perfected. (4) Physiological, biochemical and molecular mechanisms of H_2_S-induced cross-adaptation. The study on H_2_S-induced abiotic tolerance including cross-adaptation has just started, many physiological, biochemical and molecular mechanisms require being expounded using transcriptome, proteome and metabolome approaches.

## Author Contributions

Z-GL wrote and revised the paper, XM and Z-HZ provided the idea.

## Conflict of Interest Statement

The authors declare that the research was conducted in the absence of any commercial or financial relationships that could be construed as a potential conflict of interest.

The reviewer SM and handling Editor declared their shared affiliation, and the handling Editor states that the process nevertheless met the standards of a fair and objective review.
